# Supplementary material for: Comparative multiomics analysis of cell physiological state after culture in a basket bioreactor
Source: Sci Rep. 2022 Nov 23;12:20161. doi: 10.1038/s41598-022-24687-4 (PMC9686226; doi:10.1038/s41598-022-24687-4)
Supplement: Supplementary file 1 — Supplementary Information 1. [file 41598_2022_24687_MOESM1_ESM.zip › raw data/Transcriptomic raw data/7.AS/2.dasplot/TE1vsTC1/A5SS/20_CCDC9_NW_023666073.1_20320363_20320525_+@NW_023666073.1_20320363_20320781_+@NW_023666073.1_20321647_20321720_+.pdf]

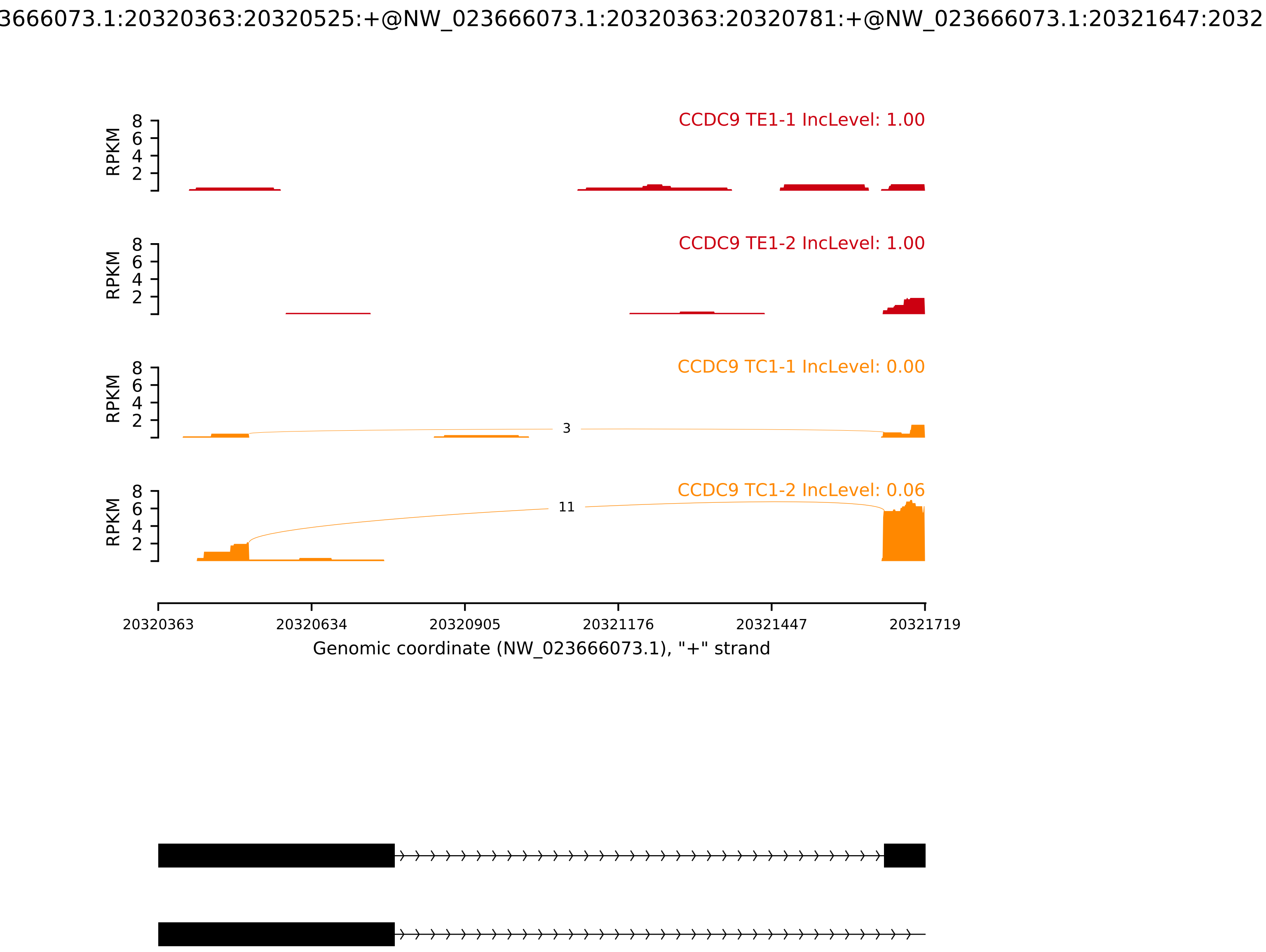
3666073.1:20320363:20320525:+@NW_023666073.1:20320363:20320781:+@NW_023666073.1:20321647:2032
CCDC9 TE1-1 IncLevel: 1.00
CCDC9 TE1-2 IncLevel: 1.00
CCDC9 TC1-1 IncLevel: 0.00
CCDC9 TC1-2 IncLevel: 0.06
RPKM
8
6
4
2
3
11
20320363
20320634
20320905
20321176
20321447
20321719
Genomic coordinate (NW_023666073.1), "+" strand
